# Improvement of Neutral Lipid and Polyunsaturated Fatty Acid Biosynthesis by Overexpressing a Type 2 Diacylglycerol Acyltransferase in Marine Diatom *Phaeodactylum tricornutum*

**DOI:** 10.3390/md11114558

**Published:** 2013-11-13

**Authors:** Ying-Fang Niu, Meng-Han Zhang, Da-Wei Li, Wei-Dong Yang, Jie-Sheng Liu, Wei-Bin Bai, Hong-Ye Li

**Affiliations:** Key Laboratory of Eutrophication and Red Tide Prevention of Guangdong Higher Education Institutes, Jinan University, Guangzhou 510632, China; E-Mails: 442359764@qq.com (Y.-F.N.); 756057321@qq.com (M.-H.Z.); 27432133@qq.com (D.-W.L.); tywd@jnu.edu.cn (W.-D.Y.); ljs@jnu.edu.cn (J.-S.L.); baiweibin@163.com (W.-B.B.).

**Keywords:** diatom, diacylglycerol acyltranferase, PUFA, neutral lipid

## Abstract

Microalgae have been emerging as an important source for the production of bioactive compounds. Marine diatoms can store high amounts of lipid and grow quite quickly. However, the genetic and biochemical characteristics of fatty acid biosynthesis in diatoms remain unclear. Glycerophospholipids are integral as structural and functional components of cellular membranes, as well as precursors of various lipid mediators. In addition, diacylglycerol acyltransferase (DGAT) is a key enzyme that catalyzes the last step of triacylglyceride (TAG) biosynthesis. However, a comprehensive sequence-structure and functional analysis of DGAT in diatoms is lacking. In this study, an isoform of diacylglycerol acyltransferase type 2 of the marine diatom *Phaeodactylum tricornutum* was characterized. Surprisingly, DGAT2 overexpression in *P. tricornutum* stimulated more oil bodies, and the neutral lipid content increased by 35%. The fatty acid composition showed a significant increase in the proportion of polyunsaturated fatty acids; in particular, EPA was increased by 76.2%. Moreover, the growth rate of transgenic microalgae remained similar, thereby maintaining a high biomass. Our results suggest that increased DGAT2 expression could alter fatty acid profile in the diatom, and the results thus represent a valuable strategy for polyunsaturated fatty acid production by genetic manipulation.

## 1. Introduction

Cell membranes are composed of proteins, phospholipids, and cholesterol. Bipolar lipids including glycerophospholipids and sphingophospholipids can form cell membranes. They can also regulate particular proteins through post-translational lipid modification, and serve as the main source of energy and messengers during cellular signal transduction. Glycerophospholipids are important for being structural and functional components of cellular membrane, in addition to precursors of various lipid mediators. Fatty acids should be activated to acyl-CoAs during the biosynthesis of glycerophospholipids [1]. Phospholipids are produced from glycerol 3-phosphate by the *de novo* pathway [2]. Diacylglycerol acyltransferase (EC 2.3.1.20) catalyzes the final committed step in triacylglyceride (TAG) biosynthesis and is considered a rate-limiting enzyme [3,4]. Diacylglycerol acyltransferase (DGAT) is one of the most intensively studied enzymes in the entire acyl lipid metabolism. DGAT has been identified and functionally characterized in a range of species, such as plants [5,6], mammals [7] and microalgae like *Chlamydomonas reinhardtii* [8,9]. Proteomic profiling of oil bodies isolated from the green microalga *C. reinhardtii* identified 19 new proteins associated with lipid metabolism and a diacylglycerol acyltransferase was included [10]. However, algae are unique in having multiple types of DGATs, and putative isoforms of DGAT identified in algae showed completely different functions [11]. Expression of a type 2 diacylglycerol acyltransferase from a diatom *Thalassiosira pseudonana* leads to incorporation of β-oxidation intermediates of docosahexaenoic acid into triacylglycerol in yeast [12]. Recently, DGAT1-like [13] and DGAT2B [14] from the marine diatom *Phaeodactylum tricornutum* have been functionally characterized in yeast; however, identification of other isoforms of DGAT and their functions in *P.* *tricornutum* remain largely unclear.

Microalgae offer a number of potential advantages for the production of value-added bioactive compounds, such as polyunsaturated fatty acids (PUFAs). Some essential PUFAs including DHA, EPA, and AA have critical physiological functions, e.g., preventing high cholesterol, myocardial infarction and improving high blood pressure, *etc.* To enhance PUFA yield in oil-producing microalgae has become very attractive at present. These high-productivity microalgal strains can be achieved by genetic engineering based on the crucial enzymes that can be targeted for fatty acid biosynthesis and molecular understanding of lipid metabolic pathways in certain microalgae [15,16].

Although the study on microalgae has become more pronounced because of the increasing interest in microalgal bioactive compounds and biofuel production, there remains very little research on genetic manipulation and understanding the function of the key enzyme for microalgae due to some technical limitations, especially a lack of efficient genetic transformation tools. In this study, we described a genetic transformation of diatom *P. tricornutum*. Molecular characterization of several enzymes involved in lipid accumulation has been conducted in mammals and plant [4,17,18,19,20], while such a study is urgently needed in diatoms for germplasm improvement. In this work, we first altered PUFA production by overexpressing DGAT2 in *P. tricornutum*.

## 2. Results

### 2.1. Sequence-Structure Analysis of DGAT2 Protein

**Figure 1:**
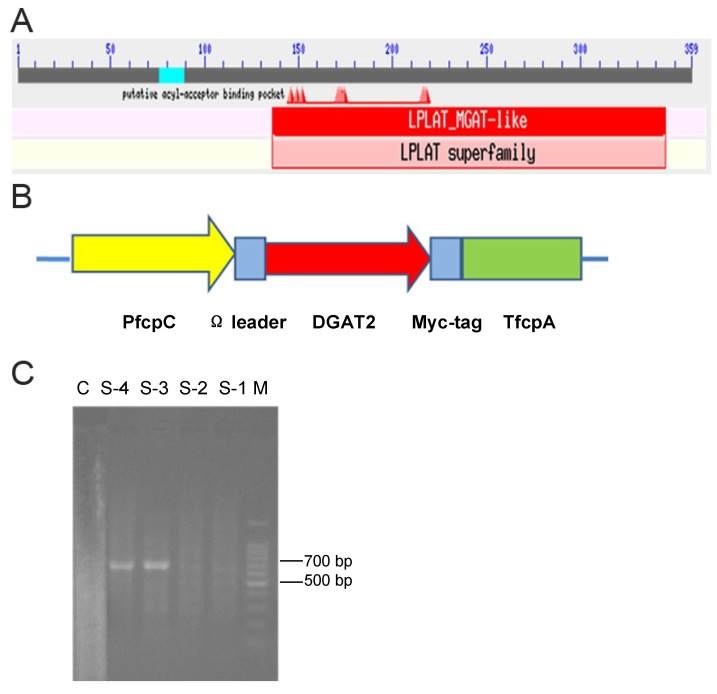
Schematic map of DGAT2 and PCR screening of transformed microalgae. (**A**) Conserved domains of DGAT2 predicted by using software CDD of the NCBI website. (**B**) Annotation of plasmid maps are given below: DGAT: diacylglycerol acyltranferases. PfcpC and TfcpA: promoter of fcpC and terminator of fcpA gene of *P. tricornutum*, respectively. (**C**) Single-cell PCR analysis of transgenic *P. tricornutum*. Lane M: 100 bp DNA ladder; lane S-1, S-2, S-3, S-4: PCR (polymerase chain reaction) of transgenic *P. tricornutum*; lane C, negative control of wild type *P. tricornutum*; a 0.7-kb band in lane S-1, S-2, S-3, S-4 indicates the expected CAT band.

Amino acid sequence of putative DGAT2 (Phatrdraft_49462, GenBank accession: XP_002184226 and AFQ23659) was analyzed on the NCBI website. Putative conserved domains were revealed, including acyl-acceptor binding pocket and LPLAT_MGAT-like domain which belongs to the LPLAT superfamily (Figure 1A).

In the transformation construct pHY31, the DGAT2 gene was cloned between the fcpC promoter and fcpA terminator of fucoxanthin chlorophyll a/c binding protein (fcp) gene of *P. tricornutum* (GenBank accession: Z24768). Moreover, the omega leader sequence and “ACC” nucleotide motif were added before DGAT2 to enhance its translation (Figure 1B).

After transformation, transgenic microalgal cells were selected and grown for at least four successive subculture cycles under chloramphenicol selection before molecular characterization. Single-cell polymerase chain reaction (PCR) was carried out to screen the transformed *P. tricornutum* for the introduced expression cassettes. PCR with primers of CAT showed that an expected 0.7 kb band was present in the transformed microalgae, while no band was present in wild type (Figure 1C). The results suggested that the pHY31 was successfully introduced into *P. tricornutum*.

### 2.2. Photosynthesis Activity and Growth of Transgenic *P. tricornutum*

**Figure 2:**
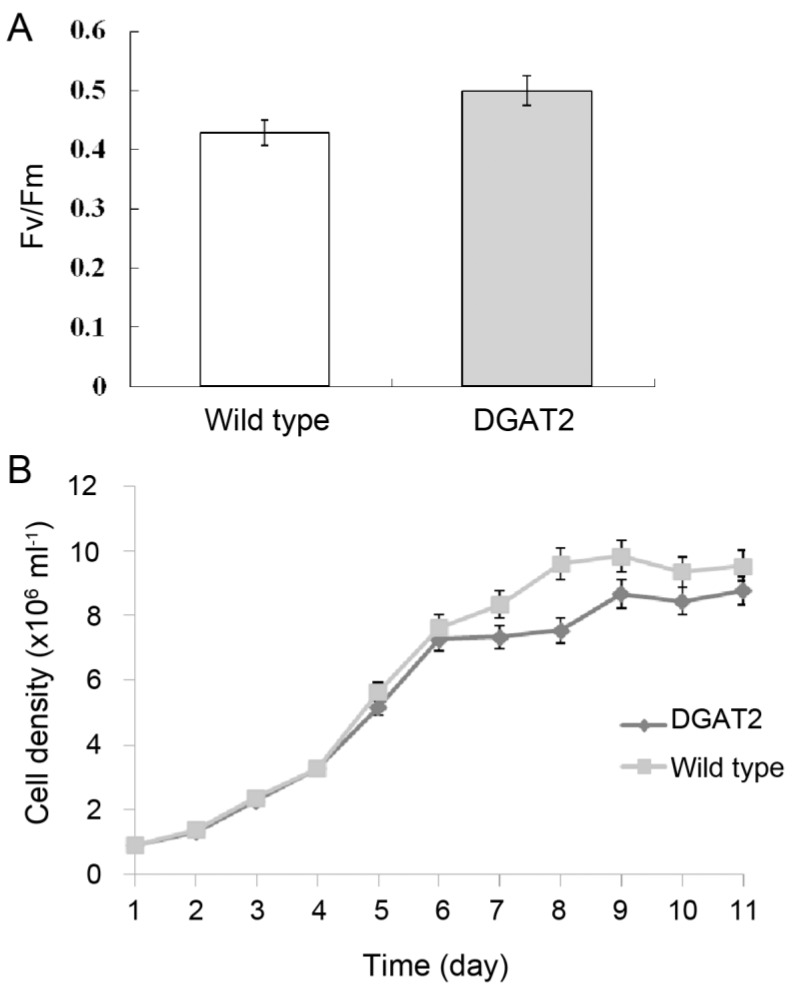
Photosynthesis activity and growth curves of *P. tricornutum*. (**A**) Photosynthesis activity; (**B**) Growth curves. DGAT2: transgenic *P.* *tricornutum*.

To examine whether the overexpression of DGAT2 had any effect on the physiological characteristics of the transgenic *P. tricornutum*, photosynthetic performance and acclimation status of diatom cells were indicated by measuring chlorophyll fluorescence parameter Fv/Fm. DGAT2 overexpressing diatom cells monitored in the stationary phase showed only a slight increase of 10% in the value of Fv/Fm compared with the wild type (Figure 2A).

To evaluate the biomass accumulation of transgenic *P. tricornutum*, the growth curves of transgenic microalgae in f/2 medium without chloramphenicol were measured (Figure 2B). The transgenic diatoms showed overall similar growth velocity compared to wild type. In particular, the transgenic cells present similar growth with wild type, while slightly lower growth during the stationary phase. The result demonstrated that transgenic *P. tricornutum* was able to grow well photoautotrophically in the medium.

### 2.3. Analysis on Lipid Content and Fatty Acid Composition

**Figure 3:**
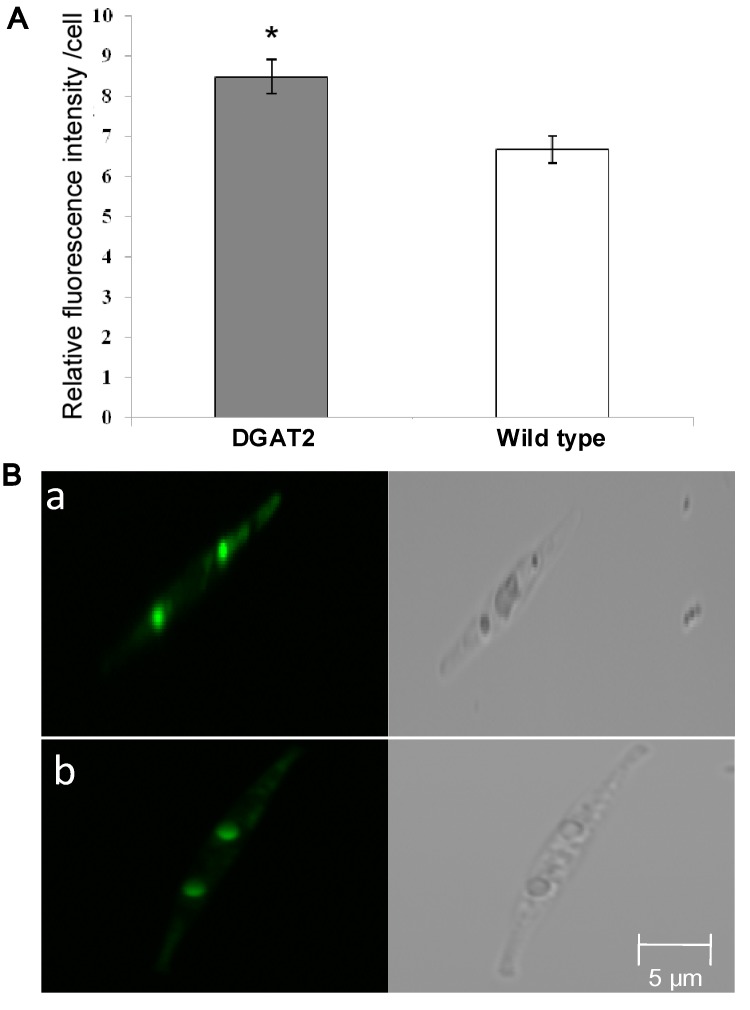
Neutral lipid content and confocal images of *P. tricornutum* cells stained with Nile red. Cells were sampled at day 7 in the stationary phase. (**A**) Relative neutral lipid content per cell determined by Nile red fluorescence. Significant change is indicated by a *p* value < 0.05 (*t*-test). (**B**) Confocal images. a, transgenic microalgae; b, wild type. Left panel: Red fluorescence of neutral lipids; right panel: DIC (differential interference contrast). Bars = 5 μm.

Diatom cells exhibited up to a 35% increase in neutral lipid content per cell measured by Nile red fluorescence staining (Figure 3A). The neutral lipid content in wild type cells was initially measured to be 27.5% in dry weight by gravimetric determination; therefore, the neutral lipid content in the dry weight of transgenic diatom cells was calculated to be 37.2%. Diatom cells were further photographed with a confocal laser-scanning microscope following Nile red staining. Transgenic microalgae exhibited a slightly higher fluorescent signal of oil bodies in the transgenic line (Figure 3B).

As shown in Table 1, an overall change in fatty acid composition between transgenic and wild type cells was the significant increase of the unsaturated fatty acids. Monounsaturated fatty acids (MUFAs) showed an increase of 35.6%, and polyunsaturated fatty acids (PUFAs) showed an increase of 34.8%. Especially the drastic increase of C20:5 (eicosapentaenoic acid, EPA) at 76.2%, with the proportion increased from 4.21% to 7.42%.

**Table 1:** Changes in fatty acid composition in transgenic microalgae.

Fatty Acid	Wild Type	DGAT2
C14:0	1.62	1.36
C15:0	0.18	0.36
C16:0	6.43	6.69
C17:0	0.1	0.05
C18:0	3.35	5.5
C20:0	0.08	1.5
C21:0	0.49	0.48
C22:0	0.05	0.15
C24:0	0.7	1.54
SUM SFA	13	17.63
C16:1	6.11	9.31
C18:1	1.03	0.29
C22:1	ND	0.04
C24:1	ND	0.04
SUM MUFA	7.14	9.68
C16:3	2.26	1.87
C18:2	0.78	0.61
C18:3	0.08	ND
C20:2	0.05	0.05
C20:5	4.21	7.42
C22:6	ND	ND
SUM PUFA	7.38	9.95

Fatty acid content is expressed as a percentage of dry biomass. Abbreviations: SFA, saturated fatty acid; MUFA, monounsaturated fatty acid; PUFA, polyunsaturated fatty acid; ND, not detected.

### 2.4. Expression of DGAT2 Determined by qPCR and Western Blot Analysis

**Figure 4:**
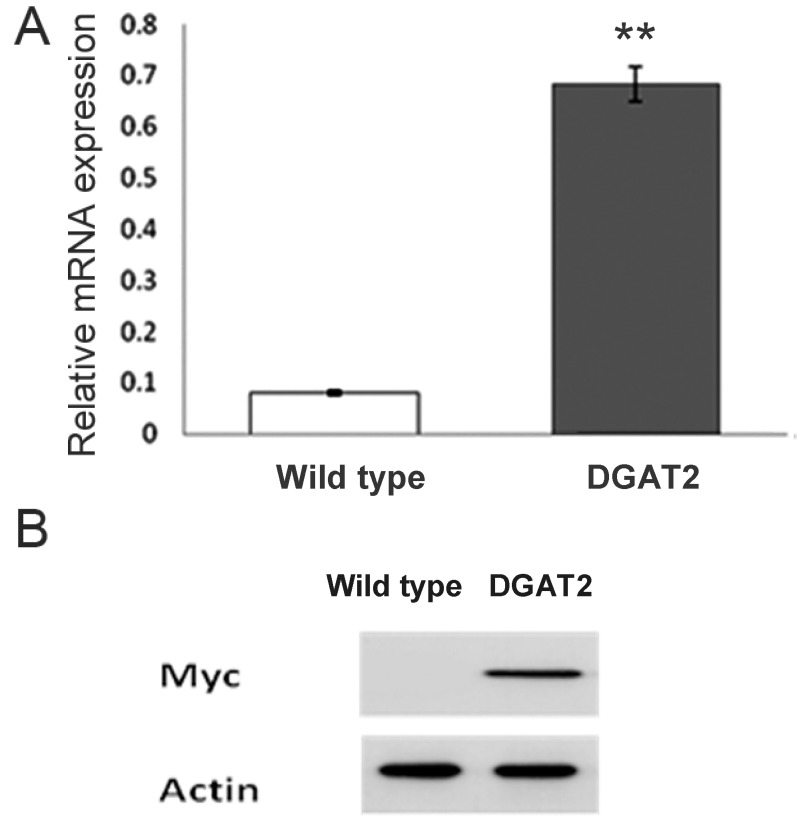
Expression of DGAT2 in transgenic *P. tricornutum.* (**A**) qPCR analysis; (**B**) Western blot analysis, DGAT2 was detected by anti-Myc antibody*.*

The transcription level of DGAT2 was determined by quantitative PCR (qPCR) in the stationary phase to observe if the mRNA levels changed at the same time. As shown in Figure 4A, the transgenic microalg ae exhibited a significant increase of 6.5-fold. It indicates that the DGAT2 overexpression had successfully enhanced the mRNA level of DGAT2 gene.

The protein level of DGAT2 was detected by Western blot analysis. As shown in Figure 4B, a cross-reacting band detected with anti-Myc antibody was present in transgenic microalgae overexpressing DGAT2 tagged with Myc, while such band was not present in wild type control. Hence, the introduced DGAT2 gene was successfully expressed at protein level.

## 3. Discussion

Studies on the key enzymes involved in lipid metabolism have been intensively conducted in some traditional model organisms of microbes, mammals and higher plants*.* However, such studies in microalgae are slow due to some technical limitations such as the lack of a transformation system. The exploit of microalgae has opened up new, broader prospects for various kinds of bio-products. The primary requirement in the microalgal bio-products industry is to have excellent microalgal strains. The ideal strain should have a combination of characteristics including fast growth, high productivity, strong resistance and suitability for large-scale cultivation. Genetic engineering is the effective approach to achieve such microalgal strains. The current study presented a trait-improved microalgal strain through this approach. In particular, a range of novel biological processes have been found in diatoms which were presumed to be acquired during evolution [21]. Diatoms are therefore also attractive for the discovery of novel metabolic pathways which are absent in other commonly studied model organisms [22].

Glycerophospholipids are important both as structural and functional components of the cellular membrane and as precursors of various lipid mediators. Even though the biosynthesis of glycerophospholipids and phospholipids were already described [1,2], functions of DGAT in diatoms such as *P. tricornutum* are still needed to be further characterized. It has been demonstrated that DGAT can affect the oil content through catalyzing the last step on the TAG biosynthetic pathway. For instance, the expression of fungal diacylglycerol acyltransferase 2 increased maize kernel oil by 26% [5]. In the current study, DGAT2 overexpression in *P. tricornutum* increased neutral lipid content by 35%, and its mRNA expression was increased by up to 6.5-fold (Figure 4).

This work also demonstrated that DGAT2 from *P. tricornutum* has multiple functions, not only in neutral lipid accumulation, but also in determination of fatty acid composition*.* In this study, DGAT2 overexpression increased PUFAs by 34.8% (Table 1). The result is in accordance with overexpression of DGAT1 gene from *T. pseudonana* in a yeast mutant strain lacking TAG synthesis where fatty acid composition was also altered by means of incorporating very-long-chain polyunsaturated fatty acids (VLCPUFA) EPA and DHA into TAGs [12]. In addition, overexpression of *Arabidopsis* DGAT, together with LEC2 genes, was able to increase lipid accumulation and shift its composition in tobacco [17].

The results here indicated that promoted DGAT2 expression in diatom would alter fatty acid metabolism. Increased PUFAs maintain the relative fluidity of cell membranes, in order to ensure the normal physiological function of cells. Besides, some functional fatty acid moieties in grease molecules such as DHA, ALA and LA have critical physiological functions, such as preventing high cholesterol and myocardial infarction or improving high blood pressure, *etc.* Increased oil content and enriched PUFAs in microalgae showed a prospective application on health care.

## 4. Experimental Section

### 4.1. Diatom Strain and Culture Conditions

The marine diatom strain *Phaeodactylum tricornutum* (CCMP2561) was purchased from the National Center for Marine Algae and Microbiota (NCMA, formerly the CCMP), USA. Diatoms were grown containing f/2 medium as batch cultures in flasks. Cultures in liquid medium or on the plate were grown at 21 ± 1 °C in an artificial climate incubator (Ningbo,China). Cool-white fluorescent tubes provided an irradiance of 200 µmol photons m^−2^ s^−1^ under long-day light conditions (15/9 h light/dark).

### 4.2. Cloning of DGAT2 and Generation of Transgenic *P. tricornutum* by Electroporation

The full-length DGAT2 coding region containing two introns was cloned by PCR with genomic DNA. Genomic DNA of *P. tricornutum* was extracted using the Universal Genomic DNA Extraction Kit Ver.3.0 (Takara, Dalian, China). The primer pairs for DGAT2 (Phatrdraft_49462) were Pt55 (5′-ACCATGAAAGAAAGAAGATCTGGCCTAA-3′) and Pt75 (5′-GTCTGTTCCAATAGTTTCAGCGTTTT-3′). DGAT2 was cloned into a *P. tricornutum* transformation vector pHY18 derived from pHY11 [23] and then transformed by electroporation following the previously developed protocol by *Niu et al*. [23]. Selected cell lines were subcultured in f/2 medium once a week supplemented with 200 mg^−1^ chloramphenicol. Cells in the stationary phase were collected for molecular characterization.

### 4.3. Selection and PCR Screening of Transgenic Microalgae

After three weeks of incubation of the plates under standard growth conditions for *P. tricornutum*, putative transformed colonies emerging on the selection medium were counted. Survived colonies were picked and inoculated into liquid f/2 medium containing 200 mg L^−1^ chloramphenicol, then passaged for five more cycles on selection medium to obtain stable transgenic microalgae.

To indicate the integration of CAT in microalgae, cells were subjected to PCR analysis with CAT primers CATf and CATr. PCR was programmed as in promoter cloning above. PCR-confirmed cultures were then subjected to successive cultures.

### 4.4. Measurement of Photosynthesis Activity

Chlorophyll fluorescence parameter Fv/Fm (ratio of variable/maximum fluorescence) is the maximum photochemical quantum yield of PSII reaction center to reflect the light energy conversion efficiency of photosynthesis. The Fv/Fm in the *P. tricornutum* culture was measured with a Handy-PEA chlorophyll fluorometer (Model AD200, Beckman Coulter, Brea, CA, USA) according to Macedo *et* *al.* [24].

### 4.5. Lipid Analysis

Lipid content in *P. tricornutum* was measured by Nile red staining according to Yang *et al.* [25]. Particularly, 30 μL of solution of Nile red (0.1 mg mL^−1^ in acetone) was added into 3 mL of cell culture for staining. Excitation and emission wavelength for detection were set at 530 nm and 580 nm, respectively. Non-stained cultures were used as an auto-fluorescence control. The relative fluorescence intensity reflects the difference of lipid content between stained cells and non-stained cells.

The morphology of microalgal cells were observed under a laser-scanning confocal microscope LSM 510 META (Zeiss, Germany). Cells were stained in the dark by mixing the Nile red (0.1 mg mL^−1^ in acetone) directly with the culture suspended in the medium in ratio of 1:100 for about 10 min. The wavelengths for oil body observation were 525 nm for excitation and 550–575 nm for emission.

Lipid composition was analyzed by gas chromatography-mass spectrometry (GC-MS) according to Yang *et al.* [25]. In brief, microalgae were collected and mixed with KOH-CH_3_OH, then subjected to ultrasonication. The supernatant was mixed with HCl-CH_3_OH, and later with *n*-hexane. Then the upper liquid was blown dry by liquid N_2_ for determination of fatty acids by GC-MS. The integrated peak areas were determined and calculated by normalization to obtain the percent contents of fatty acid composition.

### 4.6. Transcript Abundance of DGAT2 Analyzed by qPCR

Transcriptional level of DGAT2 was determined by qPCR performed in Boxin Co., Guangzhou, China. Total microalgae RNA was extracted [26] from the microalage cells and reverse-transcribed using an Omniscript reverse transcription kit (Qiagen, GmbH, Hilden, Germany) with random hexamer primers. Reactions were performed in 96-well optical reaction plates using a SYBR Green Kit (Takara, Dalian, China) and a 7300 Sequence Detection System (AB7300, Applied Biosystems, Life Technology, Carlsbad, CA, USA) following the manufacturers’ instructions. Primers used for DGAT2 were Q55 (5′-GATCTGGCCTAAATCCGTCA-3′) and Q75 (5′-CGACGATGAGACGATCAAGA-3′). β-actin was used as a housekeeping internal control. The threshold cycle (*Ct*) for each well was measured, and mRNA levels of the DGAT2 were quantified after normalization to β-actin.

### 4.7. DGAT2 Protein Expression Analyzed by Western Blot Analysis

For Western blot analysis, approximately 200 mL microalgal cells (1 × 10^6^ cells mL^−1^) were collected by centrifugation at 5000 × *g* for 10 min at 4 °C. Total protein was extracted with a Protein Extraction Kit (KeyGEN, Nanjing, China). The concentration of the protein in the supernatant was determined by using a modified Bradford protein assay (Bio-Rad, Hercules, CA, USA). Proteins were separated by sodium dodecyl sulfate-polyacrylamide gel electrophoresis (SDS-PAGE) on a 10% gel and stained with Coomassie Brilliant Blue G-250 to visualize protein bands. Another identical gel was electrotransferred to a polyvinylidene luoride (PVDF) membrane. The membrane was blocked in phosphate buffer solution (PBST) containing 5% nonfat milk at 4 °C overnight. After washing twice in PBST, the membrane was incubated with anti-Myc antibody (Abcam, Burlingame, CA, USA) at a 1:5000 dilution for 2 h at room temperature. Then the membrane was washed and incubated with a horseradish peroxidase (HRP)-conjugated secondary antibody (Kangwei, Shanghai, China) at a 1:5000 dilution for 2 h at room temperature. The blot was washed three times and developed with the tetramethylbenzidine (TMB) reagent (Beyotime, Shanghai, China).

## 5. Conclusions

In this work, an isoform of DGAT type 2 was successfully overexpressed and increased neutral lipids and PUFA content in the marine diatom *P. tricornutum*. In addition, the results indicate a possibility to increase PUFA accumulation in microalgae by using the genetic metabolic approach. This work provides further insight into the biological significance of DGAT in diatom and the results also represent a valuable tool for development of microalgal bio-products.

## Abbreviations

CAT: chloramphenicol acetyltransferase
DGAT: diacylglycerol acyltranferase
Fcp: fucoxanthin chlorophyll a/c binding protein
NCBI: National Center for Biotechnology Information
PAGE: polyacrylamide gel electrophoresis
PUFA: polyunsaturated fatty acid
TAG: triacylglyceride
